# Advancing structure modeling from cryo-EM maps with deep learning

**DOI:** 10.1042/BST20240784

**Published:** 2025-02-07

**Authors:** Shu Li, Genki Terashi, Zicong Zhang, Daisuke Kihara

**Affiliations:** 1 Department of Computer Science, Purdue University, West Lafayette, IN, U.S.A; 2 Department of Biological Sciences, Purdue University, West Lafayette, IN, U.S.A

**Keywords:** AI, artificial intelligence, cryo-EM, deep learning, structure modeling, structure validation

## Abstract

Cryo-electron microscopy (cryo-EM) has revolutionized structural biology by enabling the determination of biomolecular structures that are challenging to resolve using conventional methods. Interpreting a cryo-EM map requires accurate modeling of the structures of underlying biomolecules. Here, we concisely discuss the evolution and current state of automatic structure modeling from cryo-EM density maps. We classify modeling methods into two categories: *de novo* modeling methods from high-resolution maps (better than 5 Å) and methods that model by fitting individual structures of component proteins to maps at lower resolution (worse than 5 Å). Special attention is given to the role of deep learning in the modeling process, highlighting how AI-driven approaches are transformative in cryo-EM structure modeling. We conclude by discussing future directions in the field.

## Introduction

Cryo-electron microscopy (cryo-EM) has now established itself as the primary method of choice in structural biology [1,2]. The number of biomolecular structures determined by cryo-EM is growing rapidly, and it is expected that this number will soon exceed that of X-ray crystallography. Due to continuous advancements in instrumentation, data processing software, and experimental protocols, the achievable resolution of cryo-EM maps is gradually improving overall. However, building atomic models from cryo-EM density maps remains challenging in many cases. This difficulty mainly arises from the low resolution of some maps. When the overall resolution of a map is low, different modeling approaches may be required compared with those used for higher resolution maps. Additionally, regions of locally low resolution within a map present another challenge, as structures in these areas cannot often be reliably modeled.

Structure modeling methods have evolved over the years alongside improvements in map resolution [3]. In early years when cryo-EM could only achieve medium-to-low resolution (~5–10 Å), structure-fitting methods were actively developed. As resolution improved, main-chain tracing methods emerged, which identify locally dense regions in a map as amino acid candidates and connect them. Recently, the advent of artificial intelligence (AI), particularly deep learning, has transformed the field of cryo-EM structure modeling. Deep learning, known for its superior performance in image processing tasks, has shown remarkable success in detecting structural features and building atomic models across various resolution ranges [4].

In this review, we examine recent advances in structure modeling for cryo-EM maps, with particular emphasis on the transformative impact of deep learning methods. We categorize methods into two groups: those designed for structure modeling in maps with resolutions up to approximately 5 Å and those for low-resolution maps with resolutions worse than 5 Å. The 5 Å resolution cutoff in structure modeling is primarily empirical. At resolutions up to approximately 5 Å, amino acids, nucleotides, and atomic positions can be identified using deep learning techniques, enabling *de novo* full-atom structure modeling. However, at resolutions worse than 5 Å, identifying such key structural features becomes challenging, and modeling must rely on structure-fitting strategies [5-8]. The former group focuses on tracing density and modeling main-chain and atomic structures of proteins, while the latter requires hybrid approaches that combine protein structure prediction with structure-fitting methods. Additionally, we discuss emerging trends that hold promise for further advancing the field. The methods discussed are summarized in Table 1.

**Table 1:** Available cryo-EM structure modeling tools.

Method	Publication year	Comments	Availability	Reference
**Methods for structure modeling for maps of up to about 5 Å resolution**				
Buccaneer	2006	Initial Cα positions found via likelihood target function; fragments extended by adding residues with Ramachandran constraints.	https://www.ccp4.ac.uk/	[5]
Pathwalking	2016	Identified pseudo-atoms are connected with TSP	http://blake.bcm.edu/emanwiki/Pathwalker	[6]
MAINMAST	2018	Identified residue positions are connected to MST. Main-chain trace is constructed as paths on the MST.	https://kiharalab.org/emsuites/mainmast.php	[7]
DeepTracer	2021	Four U-Nets detect atom, secondary structure, amino acid, and backbone. Chains traced with TSP solver.	https://deeptracer.uw.edu	[8]
CR-I-TASSER	2022	Amino acid segmentation with 3D-CNN. Uses threading for chain tracing.	https://zhanggroup.org/CR-I-TASSER/	[9]
DeepMainmast	2024	Key atoms and amino acid types detected by two U-Nets. VRP solver and DP for chain tracing and sequence assignment. Fragments from AlphaFold2 models used in modeling.	https://kiharalab.org/emsuites/deepmainmast.php https://em.kiharalab.org	[10]
ModelAngelo	2024	3D-CNN for Cα detection. GNNs for chain tracing; HMM for sequence alignment.	https://github.com/3dem/model-angelo	[11]
EModelX	2024	3D U-Net for atom and amino acid type detection. Cα positions traced, and sequence assigned.	https://bio-web1.nscc-gz.cn/app/EModelX	[12]
Cryo2Struct	2024	3D transformer for atom and amino acid type detection. HMM for chain tracing.	https://github.com/jianlin-cheng/Cryo2Struct	[13]
DeepTracer 2.0	2023	3D U-Net for key atoms and nucleotide type detection. TSP for chain tracing.	https://deeptracer.uw.edu/	[14]
EMRNA	2024	3D Swin-Conv U-Net for key atoms and nucleotide type detection. Score-based sequence alignment for chain tracing.	http://huanglab.phys.hust.edu.cn/EMRNA/	[15]
**Structure-fitting methods for maps with a resolution worse than 5 Å**				
SITUS	2002	Laplacian correlation-based fitting using FFT.	https://situs.biomachina.org	[16]
UCSF Chimera (fitmap command)	2004	Overlap/cross-correlation-based local optimization starting from random initial placements.	http://www.cgl.ucsf.edu/chimera/	[17]
GMfit	2008	Each map represented with Gaussian functions and aligns them using steepest-descent to maximize correlation.	http://creativecommons. org/licenses/by-nc/2.0/	[18]
VESPER	2021	Local density gradient-based fitting. FFT to find the optimal translation.	https://kiharalab.org/em-surfer/vesper.php	[19]
EMBuild	2022	Unet ++ generates main-chain probability map. Domain-based fitting and assembling.	http://huanglab.phys.hust.edu.cn/EMBuild/	[20]
CryoAlign	2024	A map represented as a set of points. Applies point-cloud matching.	https://github.com/HeracleBT/CryoAlign	[21]
DEMO-EM2	2024	FFT-based fitting and assembly.	https://zhanggroup.org/DEMO-EM/DEMO-EM2/	[22]
DomainFit	2024	Local model-map fitting using fitmap in Chimera multiple times.	https://github.com/builab/DomainFit	[23]
DiffModeler	2024	Uses diffusion model to emphasize protein main chains in a map. Then fits models using VESPER.	https://em.kiharalab.org/algorithm/DiffModeler	[24]

Methods mentioned in the text but not available online are omitted in this table.

FFT, fast Fourier transform. TSP, traveling salesman problem. MST, minimum spanning trees. 3D-CNN, 3D convolutional neural networks. DP, dynamic programming. GNN, graph neural networks. HMM, hidden Markov model.

### Structure modeling up to about 5 Å

Building accurate protein structure models is fundamental for interpreting cryo-EM maps. Before deep learning became widely applied to protein structure modeling, several conventional methods were developed [10,13,14]. Buccaneer [14], originally designed for X-ray crystallography, automates protein chain tracing in density maps by first identifying Cα positions, extending them into chain fragments, and then growing them using Ramachandran constraints. Pathwalking [10] uses a traveling salesman problem solver to trace the protein backbone by connecting identified residue positions in a map. MAINMAST [13] identifies local peak points of the density in the map and connects them into a minimum spanning tree, which is then iteratively refined to produce a longer backbone trace. While these conventional methods are effective at directly tracing the backbone structure from the density map, they often encounter challenges when applied to low-resolution maps, where residue positions are not clearly identified. This indicated the need for more accurate backbone structure detection.

**Figure 1:**
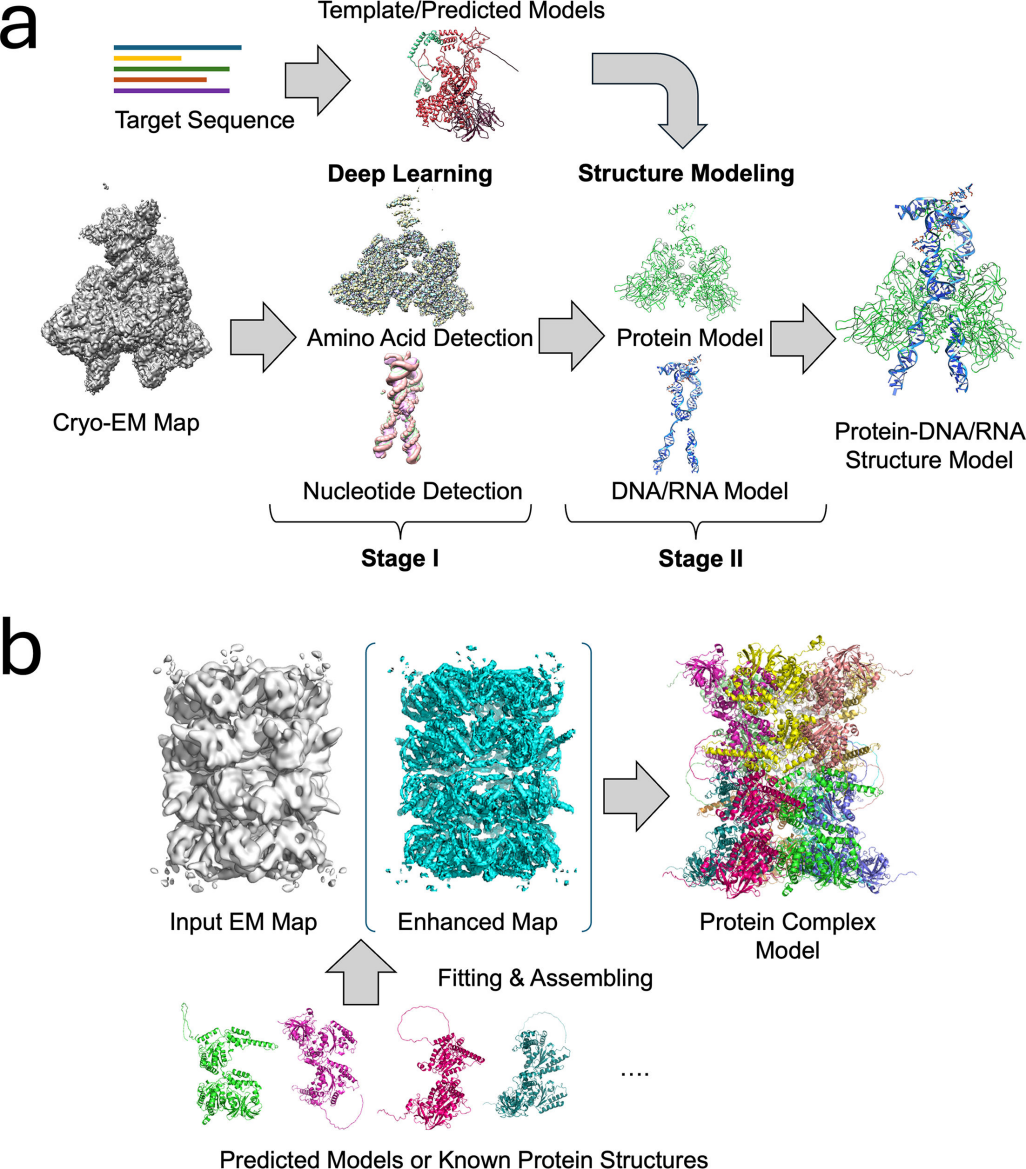
Overview of structure modeling methods for cryo-EM maps. **(a)**. Outline of protein and nucleotide structure modeling for cryo-EM maps with a resolution better than 5 Å. A two-stage strategy is adopted for most AI-based modeling methods: Stage I: For an input cryo-EM map, deep learning is used to predict amino acids, nucleotides, or key atoms. Stage II: Detected residues, nucleotides, or atoms are then connected to build a structural model. Some methods incorporate predicted (protein) structures into the modeling process. For illustration, the map EMD-7470 from the EMDB is used. **(b)** Outline of structure modeling for cryo-EM maps at 5–10 Å resolutions. At this resolution, tracing backbone structures directly from the map is challenging. Instead, modeling can be performed by fitting structural models or previously experimentally determined structures of individual proteins to the map. Some methods also use deep learning to predict or enhance protein backbones in the map before fitting. The map EMD-23192 was used for this illustration. Cryo-EM, cryo-electron microscopy.

Since 2020, deep learning approaches have revolutionized protein structure feature detection from cryo-EM maps. Unlike simple 2D image processing, cryo-EM maps are 3D data with significantly larger memory requirements and computational complexity. To address these challenges, AI-based modeling methods typically adopt a two-stage strategy that separates initial feature detection from chain tracing (Figure 1A). Deep learning methods are primarily utilized during the detection phase. These techniques enable accurate identification of the positions of key atoms, such as Cα, C, N, O, and Cβ, leading to more precise structural models of proteins. The primary focus is identifying Cα atoms, which serve as crucial anchor points for backbone tracing. Different deep neural network architectures were applied to tackle this detection task: DeepTracer [6,12] employs four individual U-Nets, which are commonly used for the image segmentation process, to predict atoms, backbone structure, secondary structure positions, and amino acid types. DeepMainmast [8], Cryo2Struct [9], and EModelX [11] implement dual U-Net architectures or their variants to detect key atoms and residue types. On the other hand, CR-I-TASSER [25] and ModelAngelo [7] use a single 3D convolutional neural network, which is a conventional architecture suitable for object detection tasks, specifically for Cα atom detection. SMARTFold [26] integrates a structure prediction pipeline similar to AlphaFold2 [15] with detected amino acid residue information from cryo-EM maps. However, the inherent characteristics of cryo-EM, such as its varying local resolution, make amino acid-type detection particularly challenging in locally low-resolution regions.

Following the deep learning-based detection stage, most methods rely on conventional algorithms for chain tracing, primarily due to the complex nature of connecting detected features into complete protein chains. These methods predominantly adopt fragment-based approaches with various optimization strategies: DeepTracer first connects Cα atoms using traveling salesmen problem (TSP) solvers and then identifies secondary structure fragments; CR-I-TASSER uses threading to identify template fragments; DeepMainmast combines vehicle routing problem solver and dynamic programming for sequence assignment, followed by fragment assembly using constraint programming; EModelX performs Cα trace sampling and Cα-sequence alignment for fragment assembly; Cryo2Struct applies hidden Markov models and Viterbi algorithm to align sequences to backbone fragments; and ModelAngelo optimizes predicted residue positions and orientations by the graph neural networks, which considers feature vectors for each residue and the density distribution around the residue. It needs to be noted that both DeepMainmast and EModelX can leverage AlphaFold-predicted structures as supplementary information to complete regions where cryo-EM density is poorly resolved, effectively combining experimental data with AI-predicted structures. Unlike conventional optimization-based methods whose runtime can be unpredictable, ModelAngelo’s deep learning approach offers more controllable computational resources and typically achieves faster processing times on GPUs.

In recent years, several methods have been extended to nucleic acid modeling. DeepTracer 2.0 [12] includes a segmentation step for separating cryo-EM maps, detecting the amino acid and nucleotide separately. CryoREAD [27] extends the DeepMainmast approach to nucleic acids. It uses a U-Net architecture to detect phosphate, sugar, and base positions to construct an atomic model of DNA/RNA. ModelAngelo has also extended its capabilities to RNA/DNA modeling. EMRNA [28] utilizes an attention-enhanced U-Net to generate probability maps for key atomic positions and nucleotide types, and then to apply a graph-based optimization strategy, incorporating a TSP-like approach.

Despite these advancements, the large size and varying dimensions of cryo-EM maps make end-to-end training of deep learning models computationally infeasible. As a result, these methods rely on a two-stage strategy, where the success of the second stage depends heavily on the quality of results from the first stage. The advantage of the two-stage process is that modeling is possible with reasonable computational resources. On the other hand, the weakness is that any errors or inaccuracies in feature detection during stage one are likely to propagate and hinder the chain-tracing process.

Although deep learning has enhanced modeling accuracy by detecting atom and residue positions not visible to the naked eye, *de novo* tracing in maps with a resolution of 5 Å or worse remains challenging. For such low-resolution maps, structure fitting approaches are often required.

### Model/map quality validation

Model quality validation is a critical final step in structure modeling from cryo-EM maps [29]. It helps to refine models and identify modeling errors. In recent years, many validation tools that use AI have been proposed [4]. Among these tools, the deep-learning-based amino-acid-wise quality (DAQ) [30] score assesses the local model quality at the residue level for protein structures built from cryo-EM maps within 2.5–5 Å resolution. DAQ assesses Cα positions, amino acid types, and the secondary structure at the Cα positions in a protein structure model. DAQ has been computed for all the Protein Data Bank (PDB) entries from cryo-EM maps determined at a resolution between 2.5  Å and 5.0  Å and stored in the DAQ-Score Database [16] (https://daqdb.kiharalab.org/). The database is updated monthly to reflect PDB and EMDB updates. DAQ scores are also accessible from entry pages in the Protein Data Bank Japan (PDBj) [17].

### Structure modeling for 5–10 Å resolution

When map resolution is worse than 5 Å, tracing backbone structures becomes nearly impossible directly from the maps. For these medium–low resolution EM maps, fitting atomic models into cryo-EM maps is a widely used approach for determining the 3D structure of macromolecules (Figure 1b). Over the past two decades, various map-model fitting methods have been developed, including SITUS [19], the fitmap command in Chimera and ChimeraX [18], VESPER [31], Gmfit [21], EM-LZerD [23], and CryoAlign [22]. These conventional methods use different map representations, such as Laplacian transforms [19], Gaussian mixture models [21], local density gradient vectors [31], 3D Zernike Descriptors [23], point clouds [22], or direct density values [18], to compute similarity and achieve optimal superimposition between the model and the cryo-EM map.

DomainFit [20] and DEMO-EM2 [24] go one step further to automatically generate a macromolecule structure model by fitting multiple subunit proteins in a cryo-EM map. DomainFit uses random sampling and optimization method from ChimeraX’s fitmap command, followed by an evaluation of the fitted domain models using *P*-values. DEMO-EM2 searches for the optimal alignment based on model–map correlation and then assembles fitted models.

Recently, it was found that the structure fitting accuracy can be improved by processing cryo-EM maps to emphasize structural information using AI-based methods before fitting. Such AI-based fitting and modeling methods, EMBuild [32] and DiffModeler [33], extract structural features from the cryo-EM map and then assemble them into protein macromolecule structures. EMBuild uses a U-Net first to predict the main-chain probability in the map. It then fits domains of protein models using a fast Fourier transform-based fitting method, which are finally assembled into a complete complex structure. DiffModeler is designed for building large macromolecular structures in cryo-EM maps at intermediate resolutions. It integrates a diffusion model, a recent generative deep neural network, for enhancing backbone regions in a map and AlphaFold2 [15] for protein structure prediction. Using a diffusion model, the DiffModeler protocol refines an initial low-resolution map into a high-resolution backbone map. Protein structures predicted by AlphaFold2 are then fitted into the refined backbone map using VESPER, which performs a global search to find the optimal aligned positions. Finally, the best-fitting subunit poses are combined to reconstruct the complete protein complex. DiffModeler achieved significantly better performance in macromolecular structure modeling compared with conventional and other AI-based methods.

The advantage of these structure-fitting methods is their ability to fit structures and model large macromolecular complexes in maps with resolutions as low as ~15 Å. The fitting procedure is less computationally demanding compared with *de novo* modeling, enabling the modeling of larger protein complexes, including those with up to 50 subunits. However, a key limitation is that the performance of these methods depends heavily on the quality of the input structure models, whether they are generated using predictive algorithms or retrieved from structural databases.

## Conclusion

The field of biomolecular structure modeling from cryo-EM maps has undergone a remarkable transformation [34,35] driven by advancements in both experimental techniques and computational methods. The integration of deep learning approaches has particularly revolutionized the ability to interpret cryo-EM density maps across various resolution ranges, making structure determination more efficient and accurate than before.

One of the most exciting frontiers in cryo-EM modeling is the development of multimodal learning approaches that can simultaneously leverage three key modalities: experimental density maps, protein sequences, and structural information. For instance, a logical extension of current modeling methods would be an integration with structure prediction methods, such as AlphaFold3 [36], which has recently made substantial advancements. Another promising direction is addressing heterogeneity in modeling [37]. Incorporating these additional sources would help resolve ambiguities, especially in regions where density maps are at lower resolutions. However, modeling approaches must carefully consider the level of information present in experimental density data and avoid overinterpreting the density.

These advances, along with enhancements in cryo-EM hardware and sample preparation, will empower structural biologists to tackle increasingly complex problems at unprecedented speeds, meeting the growing demands for high-throughput structure determination [38] while advancing our understanding of molecular machinery.

**Perspectives**

- Structure modeling is an important step for interpreting cryo-EM maps. A structure model is essential for understanding functional mechanisms of biological macromolecules.
- Modeling methods for cryo-EM can be categorized into two types, main-chain tracing for higher resolution range and structure fitting for a lower resolution range.
- AI approaches have been successfully applied to structure modeling methods. This trend is expected to continue in future developments.

## Abbreviations

AI: artificial intelligence
DAQ: deep-learning-based amino-acid-wise quality
GMMs: Gaussian mixture models
PDB: Protein Data Bank
TSP: traveling salesmen problem
VRP: vehicle routing problem
VRP: vehicle routing problem
cryo-EM: cryo-electron microscopy
